# Investigating the loco-regional control of simultaneous integrated boost intensity-modulated radiotherapy with different radiation fraction sizes for locally advanced non-small-cell lung cancer: clinical outcomes and the application of an extended LQ/TCP model

**DOI:** 10.1186/s13014-020-01555-x

**Published:** 2020-05-27

**Authors:** Bo Qiu, Qi Wen Li, Xin Lei Ai, Bin Wang, Jian Huan, Zheng Fei Zhu, Gen Hua Yu, Ming Ji, Hai Hang Jiang, Cheng Li, Jun Zhang, Li Chen, Jin Yu Guo, Yin Zhou, Hui Liu

**Affiliations:** 1 Department of Radiation Oncology, Sun Yat-sen University Cancer Center, 651 Dongfeng Road East, Guangzhou, Guangdong 510060 P. R. China; 2 State Key Laboratory of Oncology in South China, Sun Yat-sen University Cancer Center, 651 Dongfeng Road East, Guangzhou, Guangdong 510060 P. R. China; 3 Collaborative Innovation Center for Cancer Medicine, Sun Yat-sen University Cancer Center, 651 Dongfeng Road East, Guangzhou, Guangdong 510060 P. R. China; 4 Department of Radiation Oncology, The Affilicated Suzhou Science&Technology Town Hospital of Nanjing Medical University, Suzhou, Jiangsu P. R. China; 5 Department of Radiation Oncology, Fudan University Shanghai Cancer Center, Shanghai, P. R. China; 6 Department of Radiation Oncology, Zhebei Mingzhou Hospital, Huzhou, Zhejiang P. R. China; 7 Evidance Medical Technologies Inc., Suzhou, Jiangsu P. R. China; 8 Homology Medical Technologies Inc., Suzhou, Jiangsu P. R. China

**Keywords:** Locally advanced non-small-cell lung cancer, Loco-regional progression-free survival, Fraction size, Hypo-fractionation, Tumor control probability model

## Abstract

**Background:**

To investigate the loco-regional progression-free survival (LPFS) of intensity-modulated radiotherapy (IMRT) with different fraction sizes for locally advanced non-small-cell lung cancer (LANSCLC), and to apply a new radiobiological model for tumor control probability (TCP).

**Methods:**

One hundred and three LANSCLC patients treated with concurrent radiochemotherapy were retrospectively analyzed. Factors potentially predictive of LPFS were assessed in the univariate and multivariate analysis. Patients were divided into group A (2.0 ≤ fraction size<2.2Gy), B (2.2 ≤ fraction size<2.5Gy), and C (2.5 ≤ fraction size≤3.1Gy) according to the tertiles of fraction size. A novel LQRG/TCP model, incorporating four “R”s of radiobiology and Gompertzian tumor growth, was developed to predict LPFS and compared with the classical LQ/TCP model.

**Results:**

With a median follow-up of 22.1 months, the median LPFS was 23.8 months. Fraction size was independently prognostic of LPFS. The median LPFS of group A, B and C was 13.8, 35.7 months and not reached, respectively. Using the new LQRG/TCP model, the average absolute and relative fitting errors for LPFS were 6.9 and 19.6% for group A, 5.5 and 8.8% for group B, 6.6 and 9.5% for group C, compared with 9.5 and 29.4% for group A, 16.6 and 36.7% for group B, 24.8 and 39.1% for group C using the conventional LQ/TCP model.

**Conclusions:**

Hypo-fractionated IMRT could be an effective approach for dose intensification in LANSCLC. Compared with conventional LQ model, the LQRG model showed a better performance in predicting follow-up time dependent LPFS.

## Background

Concurrent chemoradiotherapy (CCRT) has long been established as the standard therapy for unresectable locally advanced non-small-cell lung cancer (LANSCLC), while the loco-regional control (LRC) and overall survival (OS) were suboptimal. Patients still experienced a loco-regional progression rate of approximately 30 ~ 40% at 3 years in most of the randomized trials [1]. After the impact of radiation dose for NSCLC was established [2], efforts to improve the LRC have been focused on increasing the total irradiation dose. A major finding in RTOG0617 was that total dose escalation of 74Gy with 2Gy per fraction failed to improve LRC or OS [3]. Therefore, hypo-fractionation radiotherapy could be another attempt to achieve better local control for LANSCLC.

Hypo-fractionation radiotherapy enables the delivery of an increased biologically effective dose (BED) without extending the overall treatment time (OTT). Image guided radiotherapy (IGRT) and intensity-modulated radiation therapy (IMRT) conform the radiation dose to the tumor and spare adjacent critical organs [4]. Simultaneous integrated boost (SIB)-IMRT has been developed recently as a dose intensification technique, which delivers different dose prescriptions to different target volumes with promising local control. However, there are limited data on hypo-fractionated SIB-IMRT concurrent with chemotherapy in LANSCLC, and the feasibility and efficacy needs to be investigated.

Other than that, setting up tumor control probability (TCP) model remained crucial for developing new fraction scheme for radiotherapy. The linear-quadratic (LQ) model provides the theoretical background for estimating TCP via the calculation of BED. Nevertheless, the conventional LQ/TCP model was proved to be ineffective in modeling the radio-biological response of hypo- and hyper-fractionated IMRT for lung cancer. We recently developed a novel LQ model incorporating cell repair, redistribution, reoxygenation, regrowth and Gompertzian tumor growth (LQRG/TCP model) to improve the modeling accuracy for the local tumor control of small cell lung cancer (SCLC) [5]. Compared with LQ/TCP model and its following modifications, LQRG which incorporates all the four “R”s of radiobiology, might be more realistic assumptions for the prediction of TCP. It is interesting to explore whether the LQRG/TCP model can as well explain the clinical effect of hypo-fractionated IMRT with different fraction sizes in the case of NSCLC. As to our knowledge, little work has been done to modeling the time dependent loco-regional progression-free survival (LPFS) in the radiation of NSCLC and use it as a theoretical foundation to interpret the fraction size dependent clinical results of the IMRT. Some authors tried to provide more clinically relevant TCP modeling of tumor response to radiotherapy in NSCLC [6–8]. However, they were mainly restricted to stereotactic body radiation therapy (SBRT) and did not incorporate the redistribution and re-oxygenation mechanisms of clonogenic cell and the interplay of radiation treatment between fractions into the LQ formalism.

This study retrospectively analyzed the LPFS of patients with LANSCLC, who were treated by SIB-IMRT concurrent with chemotherapy at different doses per fraction, and provided evidence in support of hypo-fractionated SIB-IMRT as an alternative of conventional fractionation. An recently developed extended LQ model, LQRG, was applied to account for the LPFS of the IMRT for NSCLC.

## Methods

### Acquisition of clinical data

We retrospectively reviewed the records of 103 LANSCLC patients treated from January 2012 to May 2015 in our Center. Patients included in our study had: 1.pathologically confirmed LANSCLC (stage IIIA/B); 2.treatment with SIB-IMRT and concurrent chemotherapy; 3.ECOG performance status score 0–2 and life expectancy of more than 6 months. Patients who received prior thoracic surgery or thoracic radiation, or had other coexisting malignancy were excluded. The 7th edition of American Joint Committee on Cancer staging system was used to stage the diseases. This study was approved by the ethics committee of our center. Written informed consent to use clinical data was obtained.

### Radiotherapy and concurrent chemotherapy

Patients were immobilized and simulated according to the standard protocol of lung cancer in our center [9]. Gross tumor volume (GTV) was defined as visible primary tumors and involved lymph nodes on CT and/or PET scans. The criteria of lymph node positivity included: short axis size ≥10 mm on pretreatment CT scan, reported positive on the pretreatment PET scan, or biopsy positive on mediastinoscopy or endobronchial ultrasound-guided biopsies. The GTVs were composite volumes from CT scans of all breathing phases. Clinical target volume (CTV) included the primary lung tumor with a 0.6 cm margin, the ipsilateral hilum, involved lymph nodes region and high-risk lymph nodal regions (for example, if 4R was involved, 4 L and 2R were included in CTV) (Supplementary Figure 1). The planning target volume for GTV (PTV1) and CTV (PTV2) covered the GTV and CTV with a 0.5-cm margin, respectively.

SIB-IMRT technique was used to deliver different dose prescriptions to PTV1 and PTV2 in the same treatment fraction (for example, 65Gy to PTV1 and 50Gy to PTV2 in 25 fractions). The dose and fractions were decided by planning clinic discussion and limited by normal tissue constraints. The prescription dose to PTV1 was 60 ~ 70Gy, which was the typical radical dose for LANSCLC. The prescription dose to PTV2 was 45-54Gy, which was a standard prophylactic dose. Dose constraint for organs at risk (OAR) included: the maximum spinal cord dose < 46 Gy, mean lung dose (MLD) < 20Gy and the lung volumes irradiated above 20Gy (V20) < 35%, the maximum esophagus dose < 110% of prescription dose, and V40 of the heart < 50%. The actual radiation dose to PTV was defined as the minimum dose received by 95% of PTV. Radiotherapy was delivered using daily cone beam CT image guidance. The concurrent chemotherapy were platinum-based double-agent regimen.

### Follow-up

Patients were followed up 2 months after CCRT, then every 3 months for the first 3 years and every 6 months thereafter. Routine surveillance imaging included chest, abdomen CT and brain MRI. Patients underwent bone scan when suspected for bone metastasis, and PET scan when suspected for systemic progression. Loco-regional progression, distant metastasis and survival status were recorded. LPFS was calculated from the last date of radiotherapy to the date of loco-regional progression or the date of last visit before November 30th, 2018. Infield failure was defined as recurrent tumors mapped within PTV1. Common Terminology Criteria for Adverse Events Version 4.0 (CTCAE 4.0) was used to evaluate the toxicities.

### Statistical analyses

Continues variables were presented as median and range. The survival analyses were performed using the Kaplan-Meier method. Clinical and dosimetric factors potentially predictive of LPFS were assessed using Cox proportional hazards modeling. All statistical analyses were performed with SPSS 19.0 software (IBM), and a *P* value< 0.05 was considered statistically significant.

### Tumor control probability modeling

The conventional LQ model combined with a Binomial TCP model were described in Supplementary 1. The construction of LQRG model combined with a Gaussian TCP model are briefly presented below.

The model name “LQRG” stands for the Linear Quadratic model with 4R effects and Gompertzian tumor growth. It takes account of the four “R”s effects, and add features of delayed regrowth [10] and Gompertzian tumor growth [11].

The surviving fraction (SF) of clonogenic cells is expressed as:

1 $$ SF(t)=\exp \left(-\alpha D-\beta G\left({\tau}_R\right){D}^2+\left(\frac{1}{2}{\sigma}^2\right)G\left({\tau}_S\right){D}^2+\ln 2\frac{T-{T}_k}{\tau_P}+{\left(\ln 2\frac{t}{\tau_P}\right)}^{\delta}\right) $$

where *G(τ)* is the generalized Lea-Catcheside function [11, 12], defined by Eq. 6; *τ*_*R*_ is the average DNA repair time; *τ*_*S*_ is average resensitization time; *σ* is the variance of the Gaussian distribution of random variable *α*, immediately after an acute irradiation; *T* is the treatment time; *T*_*k*_ is the delayed time for regrowth; t is the elapsed time since the end of treatment. The first and second terms describe cell killing by one-track and two-track action (and possible repair), respectively. The third term refers to intercellular diversity of radiosensitivity and resensitization [13]. The forth term refers to the delayed tumor regrowth, while the last term involving parameter *δ* refers to the Gompertzian tumor growth after radiation [14]. Although quite different phenomena, redistribution and reoxygenation do share a common outcome [15], a postirradiation increase in the sensitivity of cells that survive an initial or partial exposure. Following Brenner et al. [13], we denote this common outcome *resensitization* and incorporates these two “R”s in the third term of the exponent of SF.

2 $$ G\left(\tau \right)=\left(\frac{2}{D^2}\right)\underset{0}{\overset{\mathrm{T}}{\int }}R(u) du\underset{0}{\overset{u}{\int }}R(w)\exp \left(-\frac{u-w}{\tau}\right) dw $$

The TCP is:

3 $$ TCP(t)=1-\frac{1}{\sqrt{2\pi }}{\int}_{-\infty}^{x_0}\exp \left(-\frac{x^2}{2}\right) dx $$

with

4 $$ {x}_0=\kern0.5em \frac{\overline{K}-{K}_{cr}}{\sigma_k}\kern0.5em =\frac{SF-\raisebox{1ex}{${K}_{cr}$}\!\left/ \!\raisebox{-1ex}{${K}_0$}\right.}{\raisebox{1ex}{${\sigma}_k$}\!\left/ \!\raisebox{-1ex}{${K}_0$}\right.} $$

where *σ*_k_ is the Gaussian width for the distribution of tumor cell numbers [7]. *K*_0_ is the initial average number of clonogenic cells and *K*_*cr*_ is a critical number under which the tumor would be controlled for an individual patient [7].

BED for the LQRG model is then expressed as:

5 $$ BED=\left(D+\frac{\beta G\left({\tau}_R\right){D}^2}{\alpha }-\frac{\frac{1}{2}{\sigma}^2G\left({\tau}_S\right){D}^2}{\alpha }-\frac{\ln 2\left(T-{T}_k\right)}{{\alpha \tau}_P}\right) $$

To compare the *α/β* ratio at the same sense of the conventional LQ model, an effective *β, β*_*eff,*_ was defined for the LQRG model.

6 $$ {\beta}_{eff}= n\beta G\left({\tau}_R\right)\hbox{-} \frac{n}{2}{\sigma}^2G\left({\tau}_S\right) $$

where n was the number of fractions. With this definition of *β*_*eff,*_ the quadratic term of *D*^*2*^ in SF of Eq. 5 can be cast into the same form as in Eq. 2: -*β*_*eff*_*Dd.* This new parameter will be utilized in later comparison of α/β between the conventional LQ model and new LQRG model in a fair way.

The relations between generalized Lea-Catcheside function G(τ) and the recovery time constant τ were computed using numerical integration method. Dose and volume of GTV were extracted from the dose-volume histograms (DVHs) of radiation plans. The clinical endpoint linked to the TCP for model fitting was the time dependent LPFS. The calculated TCP(t) is fitted to the measured LPFS(t), where t is the time from the end of radiotherapy to the time of follow up. The least chi-square method was utilized in the fitting [16]. The free parameters in the function were determined by minimizing the cost function, which was the mean absolute error, using a gradient descent method. The absolute and relative fitting errors for LPFS was calculated using formulas in Additional file 1: Appendix A of our previous study [5]. The confidence intervals for estimated parameters were determined following standard procedures in nonlinear regression [17]. The maximum log likelihood and Akaike information criterion (AIC) was computed to compare the fitting goodness and synthetic quality of the models (see Additional file 1: Appendix B of our previous study [5]). In accordance with Additional file 1: Appendix A and B of [5], the minimization of the chi-square cost function, the mean absolute error (MAE), is equivalent to the maximization of log likelihood. Leave-one-out cross validation based on the time-dependent LPFS samples was performed to compare the generalization capabilities of the models. All the aforementioned calculations in tumor control probability modeling were conducted on a cloud-based clinical data service platform OncoEvidance™ v1.0 (Homology Medical, Suzhou, China, 2019) and the fitted model of LQRG was embedded in the radiobiology module of Zeus Cloud TPS™ (Treatment Planning System) v1.0 (Homology Medical, Suzhou, China, 2019).

## Results

### Patient characteristics

The median actual radiation does to PTV1 was 63.8Gy (55.6 ~ 69.3) with a median fraction size of 2.4Gy (2.0 ~ 3.1). Patients were divided, according to tertiles of fraction size, into group A (2.0 ≤ fraction size<2.2Gy, *n* = 34), group B (2.2 ≤ fraction size<2.5Gy, n = 34) and group C (2.5 ≤ fraction size≤3.1Gy, *n* = 35). Patient demographics and disease characteristics for three groups are listed in Table 1. The radiation dosimetry data are listed in Supplementary Table 1. The relative dose intensity of concurrent chemotherapy is listed in Supplementary Table 2. Sex, age, ECOG PS, histology, clinical stage, concurrent chemotherapy, as well as GTV volumes, showed similar distribution among three groups.

**Table 1:** Characteristics of patients (*n* = 103)

Characteristics	No. of patients (%)				*p value*
	All patients	Group A (*n* = 34)	Goup B (*n* = 34)	Group C (*n* = 35)	
Sex					*0.818*
Male	88 (85.4)	30 (88.2)	29 (85.3)	29 (82.9)	
Female	15 (14.6)	4 (11.8)	5 (14.7)	6 (17.1)	
Age (year)					*0.084*
Median	58	61	58	53	
Range	30 ~ 79	38 ~ 79	34 ~ 77	30 ~ 72	
ECOG PS					*0.569*
0–1	87 (84.5)	27 (79.4)	29 (85.3)	31 (88.6)	
2	16 (15.5)	7 (20.6)	5 (14.7)	4 (11.4)	
Histology					*0.315*
Squamous cell carcinoma	54 (52.4)	22 (64.7)	18 (52.9)	14 (40.0)	
Adenocarcinoma	45 (43.7)	10 (29.4)	15 (44.1)	20 (57.1)	
Adeno-squamous cell carcinoma	2 (1.9)	1 (2.9)	0 (0)	1 (2.9)	
Lymphoepithelioma like carcinoma	2 (1.9)	1 (2.9)	1 (2.9)	0 (0)	
Clinical stage (7th edition)					*0.229*
IIIA	39 (37.9)	9 (26.5)	14 (41.2)	16 (45.7)	
IIIB	64 (62.1)	25 (73.5)	20 (58.8)	19 (54.3)	
Actual radiation dose (Gy)					*0.259*
Median	63.8	63.5	63.9	64.0	
Range	55.6 ~ 69.3	57.2 ~ 65.9	59.5 ~ 69.3	55.6 ~ 68.2	
Radiation fraction size (Gy)					*< 0.001*
Median	2.4	2.1	2.4	2.8	
Range	2.0 ~ 3.1	2.0 ~ 2.2	2.2 ~ 2.5	2.5 ~ 3.1	
Overall treatment time (weeks)					*< 0.001*
Median	5.3	6.0	5.3	4.6	
Range	3.7 ~ 7.9	5.4 ~ 7.9	4.7 ~ 7.5	3.7 ~ 6.0	
Concurrent chemotherapy					*0.170*
cisplatin + Paclitaxel/Docetaxel	73 (70.9)	20 (58.8)	25 (73.5)	28 (80.0)	
cisplatin + Pemetrexed	14 (13.6)	4 (11.8)	6 (17.6)	4 (11.4)	
cisplatin + Etoposide	2 (1.9)	1 (2.9)	1 (2.9)	0	
Others	14 (13.6)	9 (26.5)	2 (5.9)	3 (8.6)	

*Abbreviations*: *ECOG PS* Eastern Cooperative Oncology Group performance status, *BED* Biological effective dose. Categories were compared using Chi-square test. Medians were compared using non-parametric test

### Loco-regional control and survival

With a median follow-up of 22.1 months (range, 5.4 ~ 64.3), the median OS was not reached. The 2-year and 5-year OS rates were 66.1 and 23.1% respectively.

The median LPFS was 23.8 months. LPFS rates dropped from 100% at time 0 to 73.9% at 1 year, 48.6% at 2 years, and 44.7% at 3 years). A total of 47 patients developed loco-regional progression.

**Fig. 1:**
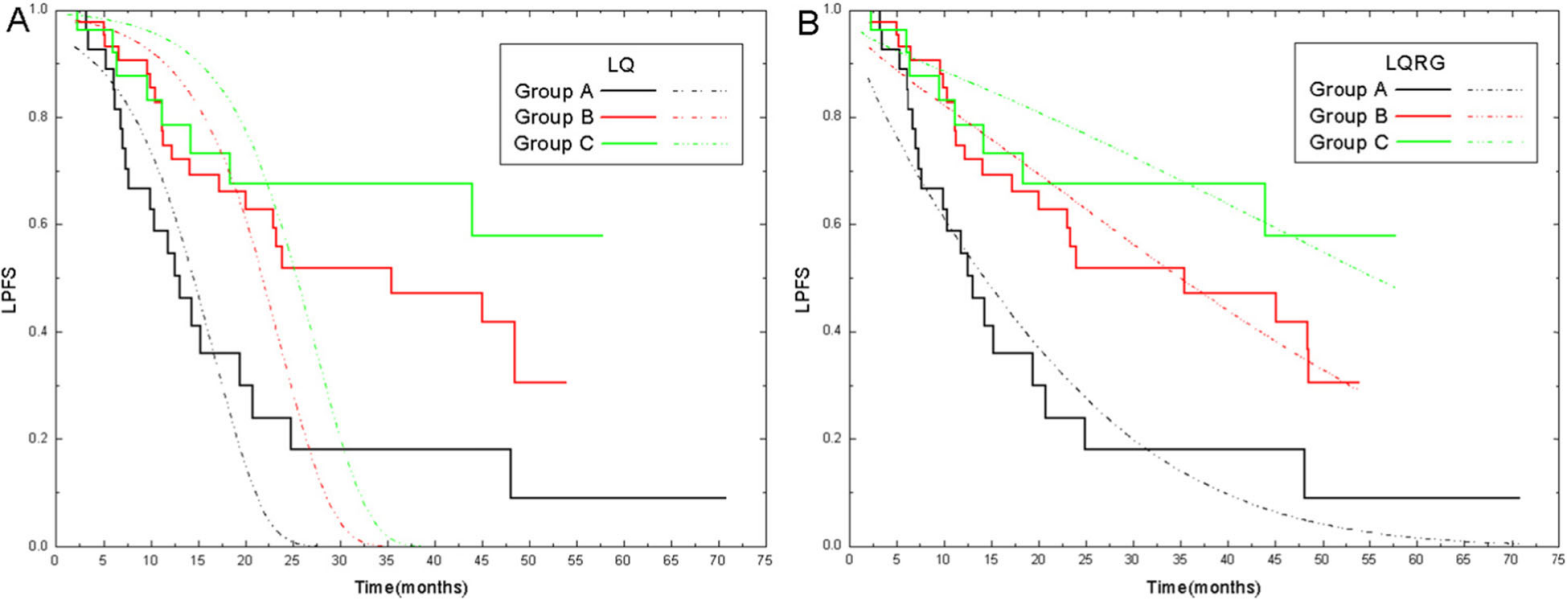
Model fitting to LPFS data using (**a**) the classical LQ and TCP models, or (**b**) the new LQRG and TCP

We assessed the impact of the following variables on LPFS: age, sex, ECOG PS, histology, tumor stage, GTV volume, radiation dose, conventional BED by LQ model, fraction size, OTT and concurrent chemotherapy (Table 2). In univariate analysis, radiation fraction size (*p* = 0.003; 2.0 ~ 2.2 vs. 2.5 ~ 3.5Gy, hazard ratio [HR] = 3.609; 2.2 ~ 2.5 vs. 2.5 ~ 3.5Gy, HR = 1.591) and OTT (*p =* 0.008, ≥5.3 weeks vs. < 5.3 weeks, HR = 2.246) were associated significantly with LPFS. Patients in group A and B had a median LPFS of 13.8 and 35.7 months, respectively. The median LPFS of group C was not reached (Fig. 1). LPFS rate at 2 years was 24.0, 55.8 and 67.8% for patients of group A, B and C, respectively. Patients with an OTT < 5.3 weeks had a longer LPFS than that with an OTT ≥5.3 weeks weeks (48.7 vs. 15.9 months).

**Table 2:** Univariate analysis of prognostic factors of loco-regional control (n = 103)

Variable	Univariate analysis	
	*p* value	HR, 95% CI
Sex (male vs. female)	0.490	0.722 (0.286 ~ 1.821)
Age (≥58 vs. < 58 yrs)	0.571	0.850 (0.485 ~ 1.491)
ECOG PS (2 vs. 0 ~ 1)	0.702	0.862 (0.404 ~ 1.842)
Histology (non-squamous vs. squamous)	0.099	1.617 (0.913 ~ 2.864)
cTNM stage (IIIA vs. IIIB)	0.703	0.893 (0.499 ~ 1.598)
GTV volume (≥78.5 vs. < 78.5 cm^3^)	0.475	1.227 (0.700 ~ 2.152)
Actual radiation dose (≥63.8 vs. < 63.8Gy)	0.575	1.175 (0.669 ~ 2.063)
Traditional BED (≥78.9 vs. < 78.9Gy)	0.134	0.648 (0.367 ~ 1.142)
Radiation fraction size	**0.003**	
(2.0 ~ 2.2 vs. 2.5 ~ 3.5Gy)	0.002	3.609 (1.572 ~ 8.286)
(2.2 ~ 2.5 vs. 2.5 ~ 3.5Gy)	0.264	1.591 (0.704 ~ 3.596)
OTT (≥5.3 weeks vs. < 5.3 weeks)	**0.008**	2.246 (1.236 ~ 4.082)
Concurrent chemotherapy	0.326	
(AP vs. TP /DP)	0.176	0.522 (0.204 ~ 1.337)
(EP vs. TP /DP)	0.562	1.809 (0.244 ~ 13.389)
(Others vs. TP /DP)	0.361	1.432 (0.663 ~ 3.091)

*Abbreviations*: *ECOG PS* Eastern Cooperative Oncology Group performance status, *BED* Biological effective dose, *ORT* Overall radiation time, *TP* Nedaplatin or cisplatin + Paclitaxel, *DP* Nedaplatin or cisplatin + Docetaxel, *AP* Nedaplatin or cisplatin + Pemetrexed, *TKI* EGFR tyrosine kinase inhibitor, *HR* hazard ratio, *CI* Confidence interval

### Patterns of failure

Seventy-five patients (72.8%) had progression of disease: 25 (24.3%) had local loco-regional progression alone, 28 (27.1%) had distant progression alone, and 22 (21.4%) had both loco-regional and distant progression. The loco-regional progression sites included ipsilateral lung (*n* = 32), mediastinum/supraclavicular lymph nodes (*n* = 6) and both (*n* = 9). There were a total of 42 (40.8%) in-field loco-regional failures and 5 (4.9%) out-field loco-regional failures.

### Toxicities

Radiation pneumonitis and esophagitis were the most common acute toxicities. Grade ≥ 3 pneumonitis and esophagitis were observed in 6 (5.7%) and 18 (17.1%) patients, respectively. There was no statistical difference in the incidence of complications among three groups. Life-threatening hemoptysis was observed in one patient irradiated at 63Gy in 21 fractions (3.0Gy per fraction) 2 months after RT. More information on toxicities are provided in Supplementary Table 3.

### Modeling

The mean total GTV doses were 62.92Gy, 64.07Gy and 62.89Gy for groups A, B and C, respectively. The mean GTV volumes were 105.83, 102.21 and 108.51cm^3^, respectively.

**Fig. 2:**
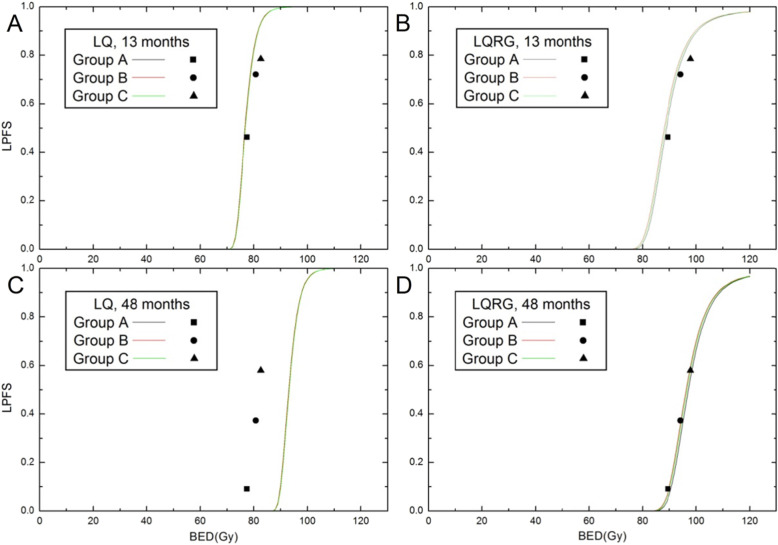
Model fitting to the LPFS data versus BED. The classical LQ and TCP models fitting to LPFS data at (**a**) 13th month and at (**c**) 48th month. The new LQRG and TCP model fitting to LPFS data at (**b**) 13th month and at (**d**) 48th month

Figure 1 presents the results of TCP (or LPFS) model fitting within a duration of 70 months. Compared with the excellent fit of the LQRG model, fitting of the LQ model failed immediately after 20 months. Using the new LQRG/TCP model, the average absolute and relative fitting errors for LPFS were 6.9 and 19.6% for group A, 5.5 and 8.8% for group B, 6.6 and 9.5% for group C, compared with the average absolute and relative fitting errors for LPFS as 9.5 and 29.4% for group A, 16.6 and 36.7% for group B, 24.8 and 39.1% for group C using the conventional LQ/TCP model. The values of model parameters and their confidence intervals are detailed in Table 3. Supplementary Table 4 lists the fitting errors of LQ model and LQRG model for the relation between LPFS and follow-up time after treatment. Figure 2 shows the BED-LPFS relation predicted at the 13th and 48th month, and suggests a better agreement between data and prediction in the LQRG model.

**Table 3:** Model Parameter Values

Parameter	LQ	LQRG	
		Value	95% CI
*ρ* (cm^−3^)	7.965 × 10^9^	2.296 × 10^9^	[1.23 × 10^9^, 4.19 × 10^9^]
*α* (Gy^−1^)	0.3932	0.06691	[0.0656, 0.0721]
*β* (Gy^−2^)	0.0429	0.1039	[0.0071, 0.1996]
*σ*^*2*^ (Gy^−2^)	/	0.1939	[0.0893, 0.2014]
*τ*_*R*_ (hour)	/	6.32	[5.51, 6.73]
*τ*_*S*_ (hour)	/	3.04	[1.12,4.99]
*τ*_*P*_ (day)	113.8	84.55	[30.1, 176.27]
*T*_*k*_ (hour)	246.06	533.5	[0.024, 1979.5]
*K*_*c*_	/	2.367 × 10^9^	[2.1 × 10^9^, 3.12 × 10^9^]
σ_k_	/	1.03 × 10^9^	[7.75 × 10^8^, 1.2 × 10^9^]
*δ*	/	0.2645	[0.2368,0.2817]
α/β in LQ and α/β_eff_ in LQRG (Gy)*	9.2	10.06	/

*LQ* Linear-quadratic model, *LQRG* LQ model incorporating cell repair, redistribution, reoxygenation, regrowth and Gompertzian tumor growth

Supplementary Table 5 lists the intermediate results of the calculation of LPFS at the end of the treatment. These intermediate items were extracted from the exponent part of the SF from both models. Supplementary Figure 2 shows the relation between generalized Lea-Catcheside function G(τ) and the recovery time constant τ for both schemes. Table 4 presents the metrics characterizing the quality of the LQ and LQRG models.

**Table 4:** Model Quality Metrics

Number of samples	LQ	LQRG
	103	103
Number of parameters	5	11
Maximum log likelihood	1.07	117.14
Akaike Information Criterion	7.86	−212.27
Average prediction accuracy in leave-one-out cross validation (%)	64.7%	88.2%

*LQ* Linear-quadratic model, *LQRG* LQ model incorporating cell repair, redistribution, reoxygenation, regrowth and Gompertzian tumor growth

## Discussion

The standard radiation scheme for LANSCLC consists of a total dose of 60 ~ 70Gy delivered in a fraction size of 2Gy per day. Various treatment schemes have been tried in an effort to improve local control by either dose escalation or shortening the total treatment time through unconventional fractionation [18]. The results from RTOG 0617 suggested that the balance between treatment-related toxicities and dose escalation must be taken into account seriously [3]. Asian patients physically have smaller lungs volume than western patients, which make it hard to escalate the total dose beyond 70Gy. Therefore, we tried to use SIB-IMRT technique to escalate the fraction size. Our results showed that in the range of 60 ~ 70Gy, total radiation dose was not prognostic of LPFS. LPFS was improved by the escalation of radiation fraction size (HR, 0.590; CI, 0.348 ~ 1001). It implied that hypofractionated SIB-IMRT could be an alternative of conventional fractionation.

Data on the efficacy of hypo-fractionated radiotherapy for LANSCLC remain limited. A systematic review of the literature about radical-intent hypo-fractionated RT for LANSCLC included 33 studies [19]. The RT dose ranged from 45.0 to 85.5 Gy, and was delivered at 15 ~ 35 fractions at a dose per fraction ranging from 2.3 to 3.5Gy. OS was found to be associated with tumor BED. A phase II study showed that escalating radiation dose to the FDG-avid tumor detected by midtreatment PET provided a favorable LRC in stage II or III NSCLC [20]. RT was delivered in 30 daily fractions up to a total physical dose of 86Gy. It achieved a promising 2-year infield LRC rate of 82% and overall LRC rate of 62%. Our study compared the LRC of different radiation fraction size and showed that hypo-fractionation (over 2.5Gy per fraction) achieved a longer LRC time than conventional fractionation. The possible advantage of hypo-fractionation includes the escalation of BED and shortening of treatment time, which is believed to limit tumor repopulation [21]. A work by Machtay et al. examined the relationship between BED and treatment outcome in conventionally fractionated and hyper-fractionated regimens, where a 1Gy increase in BED resulted in a 3% improvement in LPFS [22]. It has been reported that there is a loss of absolute 3-year survival rate of 1.6% per day OTT prolongation beyond 6 weeks [23]. Consistent with these previous findings, our data showed that fraction size and OTT were associated significantly with LPFS, and fraction size turned out to be an independent prognostic factor.

Calculated by the classical LQ model, the order of the BEDs of all three patient groups correctly predicted the height order of the LPFS curves, but the overall fitting of LPFS for NSCLC patients from follow-up data failed again just as in the case of SCLC, thereby reaffirming its incapability of TCP prediction. The LQRG and the new TCP model, however, succeeded in fitting and predicting the follow-up time dependent LPFS, which, as pointed out in the case of SCLC [5], contributed mainly to two model improvements: namely, one accounting for the 4R effect of radiobiology and the other for more reasonable assumptions for the clonogenic regrowth and cell number distribution.

As in the work on SCLC [5], an additional analysis of the intermediate results of the SF and BED further confirmed the previously revealed mechanisms behind the LQRG models, namely the positive difference between the three schemes made by the resensitization term, the slowing down of the rapid decaying rate of the LPFS as time proceeded on by the Gompertzian term and the reformulation of TCP with *K*_*cr*_, and the characteristic distinctions between the three schemes caused by the gaps among the three curves of Lea-Catchside function. Once again, these distinctions from Lea-Catchside function were embodied in the second and third term of the SF in (Eq. 5) and subtly balanced by other factors to contribute to the final comparative clinical results. For example, with the optimized value of the τ_R_ = 6.32 h, G (τ_R_) in LQRG model were 0.036, 0.040 and 0.049 for groups A, B and C, respectively, which were larger than those corresponding values of 0.033, 0.037 and 0.045 in conventional LQ model. This implied the stronger cell killing effect by two-track actions in LQRG model which might have more accurately represented the interplay between dose fractions through its precise numerical calculation of Lea-Catchside function. Moreover, the models from both conventional LQ and LQRG revealed the subtle effect of the duration of treatment through the term representing the delayed regrowth of the tumor *ln2(T-T*_*k*_*)/τ*_*P,*_ which slightly favored short treatment duration with larger fraction size in hypo-fractionated IMRT as had been anticipated in clinical settings. Consequently, the BED of the LQRG model for groups A, B and C were 85.93Gy, 91.03Gy and 95.35Gy respectively, which was in good agreement with the clinical observation that the 2.5–3.1Gy per fraction concurrent with chemotherapy showed the best LPFS. Compared with the BED of the conventional LQ model, the relative difference between the BEDs was amplified in LQRG model, which might better explain the additional long-term benefit for schemes with larger fraction sizes.

A close examination of the optimized model parameters revealed that they fell well into the published data range. The *α* was 0.067, close to the value for NSCLC in previous study [7]. To comparatively evaluate the value of *β* with published data using conventional LQ model, we recast the Eq. 5 into the form of LQ model by introducing an effective parameter *β*_*eff*_ in Eq. 6. Rather than compute the *α/β* ratio as in conventional LQ model, we computed the *α/β*_*eff*_ ratio in LQRG model to compare with its conventional counterpart, *α/β*. The optimized value of *α/β*_*eff*_ ratio in LQRG model was 10.06, which agreed well with the published consensus of around 10 for NSCLC [24]. The cell number doubling time τ_*p*_ was 84.55 days, closed to the clinically agreed value of nearly 3 months for NSCLC [7, 25]. The delay regrowth time *T*_*k*_ is 533.5 h or 22.2 days, close to the clinically agreed value of nearly 1 months [7, 25]. The repair and resensitization time constants were 6.32 h and 3.04 h respectively, within the range of fitted laboratory-based experimental data from 1 h to tens of hours [13].

The preferred log likelihood of LQRG model was 117.14, which was slightly higher than that of LQG (100.52) and substantially higher than that of LQ (1.7), indicating the reasonable improvements of the fitting capability of LQRG model over those of LQG and LQ. In order to evaluate the risk of overfitting for LQRG model with six more variables than that of conventional LQ and TCP model, AIC was developed for all the three models. AIC, which acts as an operational way of trading off the complexity of an estimated model against how well the model fits the data, is a measure of the fitting quality of an estimated statistical model, with lower value indicating higher model quality. AIC not only rewards goodness of fit, but also penalizes the number of estimated parameters which is closely related to overfitting. The fact that AIC of the fitting of LQRG model to the data was − 212.27, slightly lower than that of LQG model (− 183.03) and substantially lower than that of LQ model (7.86) validated LQRG’s superior synthetic model quality with reasonably low overfitting risk. In addition, leave-one-out cross validation for the three models showed 88.2% average prediction accuracy for LQRG model, with 85.6% for LQG and 64.7% for LQ respectively. This result of leave-one-out cross validation again demonstrated LQRG’s sound predictive power and a better generalization capability with acceptable overfitting risk. Compared to conventional LQ and TCP model, although six additional variables were added to the new models, the mechanistically driven nature of these variables could potentially interpret the outstanding balance between fitting accuracy and generalization capability. As to our knowledge, this is the first theoretical work to accurately predict the follow-up time dependence of LPFS for NSCLC with hypo-fractionated SIB-IMRT. Since the proposition of LQRG model in SCLC [5], this work has applied the same LQRG modelling framework in a different type of lung cancer -- NSCLC, and confirmed its universality in follow-up time dependent LPFS modelling for lung cancer. In this study, death was not considered as an event in the LPFS estimation if the patient was not dead of loco-regional progression. Distant metastasis was the main pattern of failure that affected survival in locally advanced NSCLC [26]. Our data showed 50 patients (48.5%) developed distant metastasis during follow-up. Therefore, considering death as an event in the LPFS might overestimate the loco-regional failure and affect the following model fitting.

This study was limited by its retrospective nature. The dose and fractions were decided by planning clinic discussion and limited by normal tissue constraints. The prescription might have been influenced by doctor’s preference, tumor volume, performance status and pulmonary function. Some factors, such as tumor volume and PS, might influence the local control. Therefore, we included these potential confounding factors in univariate and multivariate analysis to minimize the confounding effect. This study was also limited by the lack of prospective collection of toxicity data. There might be an underestimation of toxicity. Therefore, dose escalation through hypo-fractionated SIB-IMRT should always be performed with caution of toxicity. This study was also limited by a modest sample size and events which might cause overfitting of the model. A larger sample size with a longer follow-up time is warranted.

## Conclusions

Our study shows improved loco-regional control in patients treated with an escalated fraction size in the setting of concurrent chemotherapy. It supported that, for Asian patients, hypo-fractionated SIB-IMRT could be a feasible and effective approach for dose intensification in LANSCLC. Compared with conventional LQ model, the LQRG model showed a better performance in follow-up time dependent LPFS modeling. A larger sample size and longer follow-up time was warranted before widely applying hypo-fractionated RT to LANSCLC in clinical practice.

## Supplementary information

**Additional file 1.**

## Data Availability

The datasets used and/or analyzed during the current study are available from the corresponding author on reasonable request.

## Abbreviations

LPFS: Loco-regional progression-free survival
IMRT: Intensity-modulated radiotherapy
LANSCLC: Locally advanced non-small-cell lung cancer
TCP: Tumor control probability
LQRG: Linear-quadratic model incorporating cell repair, redistribution, reoxygenation, regrowth and Gompertzian tumor growth
LQ: Linear-quadratic
LRC: Loco-regional control
OS: Overall survival
BED: Biologically effective dose
OTT: Overall treatment time
IGRT: Image guided radiotherapy
(SIB)-IMRT: Simultaneous integrated boost intensity-modulated radiotherapy
SCLC: Small cell lung cancer
SBRT: Stereotactic body radiation therapy
GTV: Gross tumor volume
CTV: Clinical target volume
PTV: Planning target volume
CT: Computed tomography
PET: Positron emission tomography
MRI: Magnetic resonance imaging
SF: Surviving fraction
DVH: Dose-volume histograms
AIC: Akaike information criterion
CCRT: Concurrent chemoradiotherapy
