# Supplementary material for: Investigating the loco-regional control of simultaneous integrated boost intensity-modulated radiotherapy with different radiation fraction sizes for locally advanced non-small-cell lung cancer: clinical outcomes and the application of an extended LQ/TCP model
Source: Radiat Oncol. 2020 May 27;15:124. doi: 10.1186/s13014-020-01555-x (PMC7251706; doi:10.1186/s13014-020-01555-x)

**Supplementary 1. The classical LQ and TCP models**

The surviving fraction (SF) of clonogenic cells is expressed as:

| $SF(t)=\exp\left(-\alpha D-\beta Dd+\frac{\ln2\left(T+t-T_{k}\right)}{\tau_{P} }\right)$ | (Eq. 1) |
| --- | --- |

where the parameter *α* and *β* represent lethal lesions made by one- or two-track actions, respectively; *D* is the total doses; d is the fractional doses; *τ_P_* is the tumor doubling time; *T* is the treatment time; *T_k_* is the delayed time for regrowth; *t* is the elapsed time from the end of treatment to the time of follow up. The BED is expressed as [32]:

| $BED=D+\frac{\beta Dd}{\alpha}-\frac{\ln2\left(T-T_{k}\right)}{\alpha\tau_{P}}$ | (Eq. 2) |
| --- | --- |

The TCP is defined as the probability of killing all the clonogenic cells at instant t:

| $TCP(t)=\text{(}1-SF(t)\text{)}^{K_{0}}$ | (Eq. 3) |
| --- | --- |

where *K*_0_ is the initial average number of clonogenic cells.

| $K_{0}=V_{0} \rho$ | (Eq. 4) |
| --- | --- |

where *V*_0_ is the initial average tumor volume; *ρ* is the number of tumor cells in a unit volume.

**Supplementary 2. APPENDICES from our previous work** (*Li Q-W, Qiu B, Wang B, Zhang J, Chen L, Zhou Y, et al. Comparison of hyper- and hypofractionated radiation schemes with IMRT technique in small cell lung cancer: Clinical outcomes and the introduction of extended LQ and TCP models. Radiotherapy and Oncology. 2019;136:98-105.*)**.**

**Appendix A**

Given a collection of independent and identically distributed observation points {(x_1_,y_1_),..., (x_n_,y_n_)}, and a model described as $\tilde{y}=f\left(x\right)$, the average absolute and relative fitting errors for LPFS, i.e MAE (mean absolute error) and MRE (mean relative error), are defined as follows.

, and

.

**Appendix B**

Given a collection of independent and identically distributed observation points {(x_1_,y_1_),..., (x_n_,y_n_)}, the maximum likelihood of this observation is formulated as [33]:

$$\hat{L}=\max_{\theta\in\Theta} \prod_{i} P_{Y|\text{X}}\left(y_{i}|x_{i};\theta\right)=\max_{\theta\in\Theta} \prod_{i} \frac{1}{\sqrt{2\pi\sigma^{2}}}\exp\left\{-\frac{\left[y_{i}-f\left(x_{i};\theta\right)\right]^{2}}{2\sigma^{2}}\right\}$$

where f( x; θ) = y - ɛ denotes the fitting function with the parameter θ and the probabilistic distribution model for the noise ɛ is Gaussian with variance σ^2^. If the fitting function is of $k$ independent variables, the variance can be estimated as follows:

$\sigma^{2}=\frac{1}{n-k}\sum_{i=1}^{n} \left(y_{i}-f\left(x_{i};\theta\right)\right)^{2}$ .

Therefore, the log likelihood takes the following form:

Then the Akaike Information Criterion [34] is formulated as:

$$AIC=2k-2\ln\left(\hat{L}\right)()$$

where k is the number of model parameters.

**Supplementary Table 1**. Radiation dosimetry (n=103).

|  | All patients (n=103) | Group A  (n=34) | Group B  (n=34) | Group C  (n=35) | *p-value* |
| --- | --- | --- | --- | --- | --- |
| Dose per fraction (Gy) | 2.0~3.1 | 2.0~2.2 | 2.2~2.5 | 2.5~3.5 | *<0.001* |
| Actual radiation dose (Gy) | 63.8,  55.6~69.3 | 63.5,  57.2~65.9 | 63.9,  59.5~69.3 | 64.0,  55.6~68.2 | *0.259* |
| Mean lung dose (Gy) | 18.6, 10.6~20.8 | 18.3,  11.2~19.4 | 18.7  12.9~19.5 | 19.2  10.6~20.8 | *0.511* |
| Dmax of esophagus (Gy) | 63.9, 29.0~76.7 | 65.5,  29.0~76.7 | 64.8,  56.7~75.3 | 62.8,  37.5~68.7 | *0.779* |
| V50 of esophagus (%) | 37.1, 0.0~82.0 | 36.0,  0.0~82.0 | 46.8,  25.0~75.0 | 25.1,  0.0~69.2 | *0.347* |
| Dmax of spinal cord (Gy) | 40.0, 20.7~45.7 | 41.2,  20.7~45.7 | 39.4,  24.9~42.1 | 38.7,  21.6~41.8 | *0.217* |
| V40 of heart (%) | 7.6,  0~40.9 | 6.9  0~45.6 | 12.4  0~38.9 | 8.0  0~33.3 | *0.323* |

All values are presented as median and/or range. Medians were compared using non-parametric test.

Abbreviations: GTV= gross tumor volume; BED = biological effective dose; V(X) = the percentage of volume irradiated ＞ X Gy; Dmax = maximum dose.

**Supplementary Table 2**. Relative dose intensity of concurrent chemotherapy (n=103).

|  | Relative dose intensity % | | | | *p*-value |
| --- | --- | --- | --- | --- | --- |
|  | All patients (n=103) | Group A  (n=34) | Group B  (n=34) | Group C  (n=35) |  |
| Median | 100 | 100 | 100 | 100 | *0.843* |
| Range | 85.1~100 | 88.0~100 | 85.1~100 | 87.5~100 |  |

Medians were compared using non-parametric test.

**Supplementary Table 3**. Toxicity profiles (n=103).

| Toxicities | All patients (n=103) | Group A  (n=34) | Group B  (n=34) | Group C  (n=35) | *p*-value* |
| --- | --- | --- | --- | --- | --- |
| Pneumonitis |  | |  |  | *0.500* |
| Grade 1-2 | 42(40.8) | 16 (47.1) | 11 (32.4) | 15 (42.9) |  |
| Grade ≥3 | 6(5.8) | 3 (6.5) | 1(5.6) | 2(4.3) |  |
| Esophagitis |  |  |  |  | *0.098* |
| Grade 1-2 | 60(58.3) | 21(52.2) | 22(61.1) | 17(60.9) |  |
| Grade ≥3 | 18(17.5) | 6(13.0) | 7(19.4) | 5(14.3) |  |
| Nausea |  |  |  |  | *0.336* |
| Grade 1-2 | 13(12.6) | 5(14.7) | 2(5.9) | 6(17.1) |  |
| Grade ≥3 | 0 | 0 | 0 | 0 |  |
| Neutropenia |  |  |  |  | *0.291* |
| Grade 1-2 | 23(22.3) | 8(23.5) | 10(29.4) | 5(14.3) |  |
| Grade ≥3 | 4(3.9) | 2(5.9) | 2(5.9) | 0 |  |
| Anemia |  | |  |  | *0.514* |
| Grade 1-2 | 38(36.9) | 15(44.1) | 12(35.3) | 11(31.4) |  |
| Grade ≥3 | 1(1.0) | 0 | 1(2.9) | 0 |  |
| Hemoptysis |  |  |  |  | *0.665* |
| Grade 1-2 | 5(4.9) | 1(2.9) | 2(5.9) | 2(5.9) |  |
| Grade ≥3 | 1(1.0) | 0 | 0 | 1(2.9) |  |
| Skin toxicity |  |  |  |  | *0.502* |
| Grade 1-2 | 38(36.9) | 10(29.4) | 13(38.2) | 15(44.1) |  |
| Grade ≥3 | 0 | 0 | 0 | 0 |  |

* Chi-Square test was used to compare the toxicity among dose groups. Toxicities were graded by CTCAE version 4.0. Toxicities were recorded within 90 days of completion of radiotherapy. Pneumonitis were recorded within 1 year after radiotherapy.

**Supplementary Table 4.** The average absolute and relative fitting errors for LPFS

| **Error type** | **LQ** | | | **LQRG** | | |
| --- | --- | --- | --- | --- | --- | --- |
|  | **Group A** | **Group B** | **Group C** | **Group A** | **Group B** | **Group C** |
| Absolute | 9.5% | 16.6% | 24.8% | 6.9% | 5.5% | 6.6% |
| Relative | 29.4% | 36.7% | 39.1% | 19.6% | 8.8% | 9.5% |

BED: biological effective dose, LPFS: Local progression-free survival, LQ: linear-quadratic, LQRG: LQ model incorporating cell repair, redistribution, reoxygenation, regrowth and Gompertzian tumor growth.

**Supplementary Table 5.** Model Intermediate Items

| **Item** | **LQ** | | | **LQRG** | | |
| --- | --- | --- | --- | --- | --- | --- |
|  | **Group A** | **Group B** | **Group C** | **Group A** | **Group B** | **Group C** |
| *BED* | 77.0149 | 80.3938 | 82.315 | 85.9368 | 91.0297 | 95.3506 |
| $-\alpha D$ | -24.7409 | -25.1911 | -24.7265 | -4.3983 | -4.4783 | -4.3957 |
| $-\beta G\left(\tau_{R} \right)D^{2}$ | -5.66162 | -6.52172 | -7.71136 | -14.8864 | -17.1452 | -20.2653 |
| $\left(\frac{1}{2}\sigma^{2} \right)G\left(\tau_{S} \right)D^{2}$ | / | / | / | 13.214 | 15.2214 | 17.9979 |
| $\ln2\frac{T-T_{k}}{\tau_{P} }$ | 0.120268 | 0.101996 | 0.071543 | 0.0637 | 0.0391 | -0.0019 |
| $G\left(\tau_{R}\right)$* | 0.0333 | 0.037 | 0.0455 | 0.0362 | 0.0402 | 0.0493 |
| $G\left(\tau_{S}\right)$ | / | / | / | 0.0344 | 0.0383 | 0.047 |

n: number of fractions; LQ: linear-quadratic model LQRG: LQ model incorporating cell repair, redistribution, reoxygenation, regrowth and Gompertzian tumor growth. *: this term will be reduced to 1/n in LQ model.

**Supplementary Figure 1.** A, When station 4R was involved, the CTV encompassed adjacent 4L. B, When station 5 was involved, the CTV encompassed adjacent 4L. Red line: the contour of GTV. Pink line: the contour of CTV,

**A.**
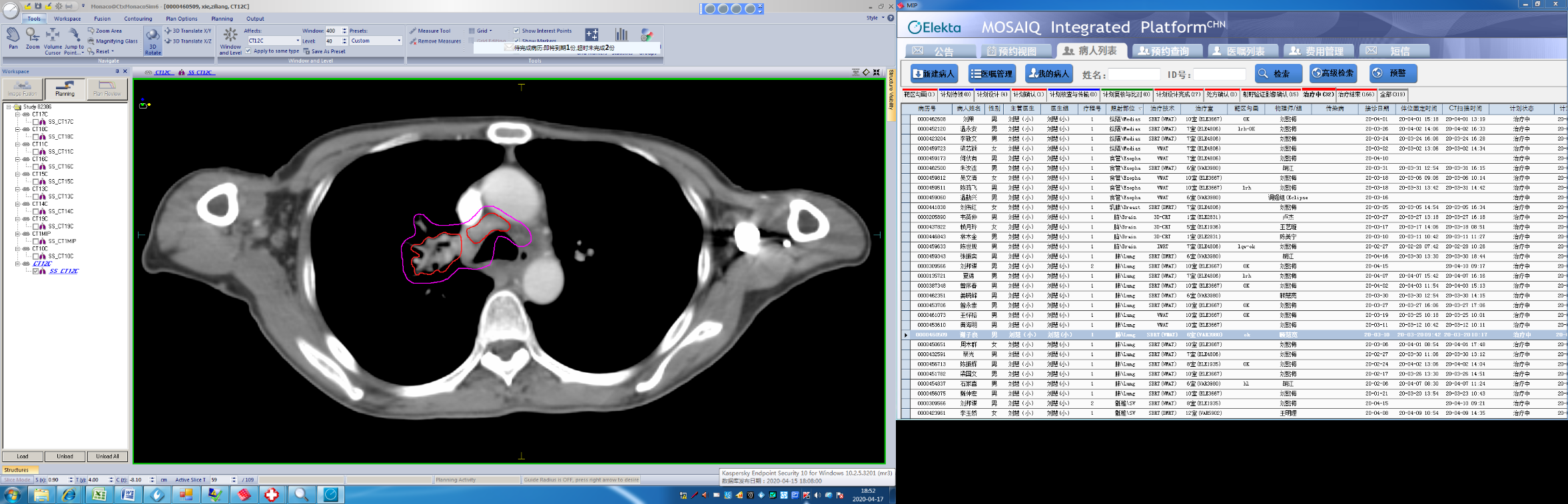


**B.**
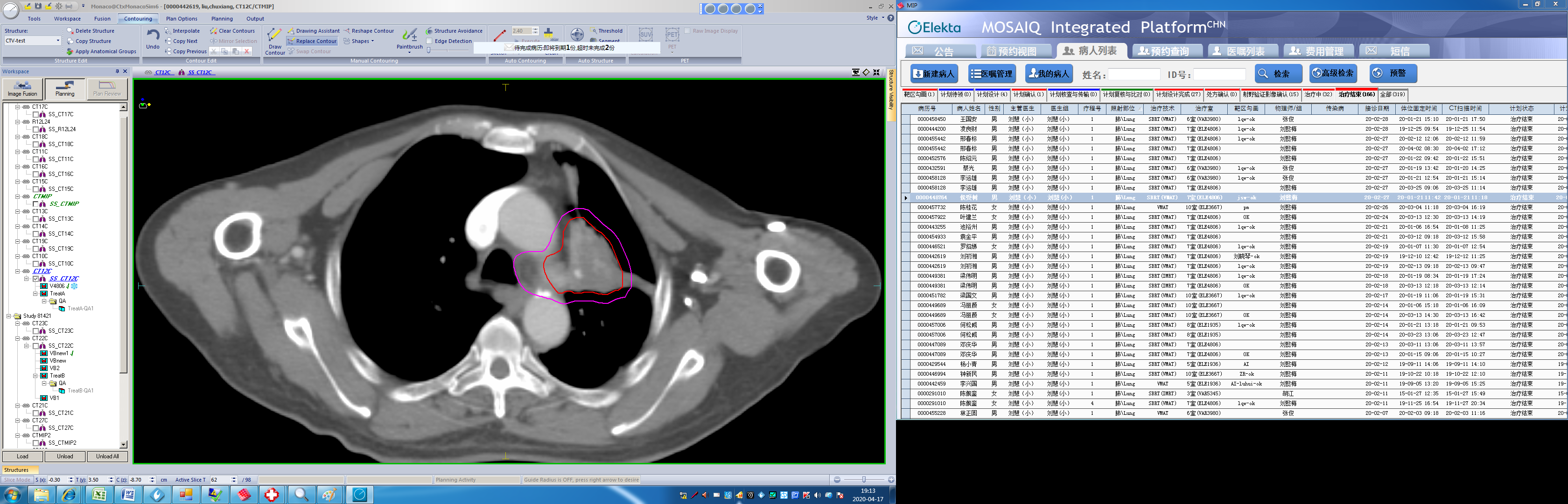


**Supplementary Figure 2.** The relation between generalized Lea-Catcheside function G(τ) and the recovery time constant τ for LQ and LQRG


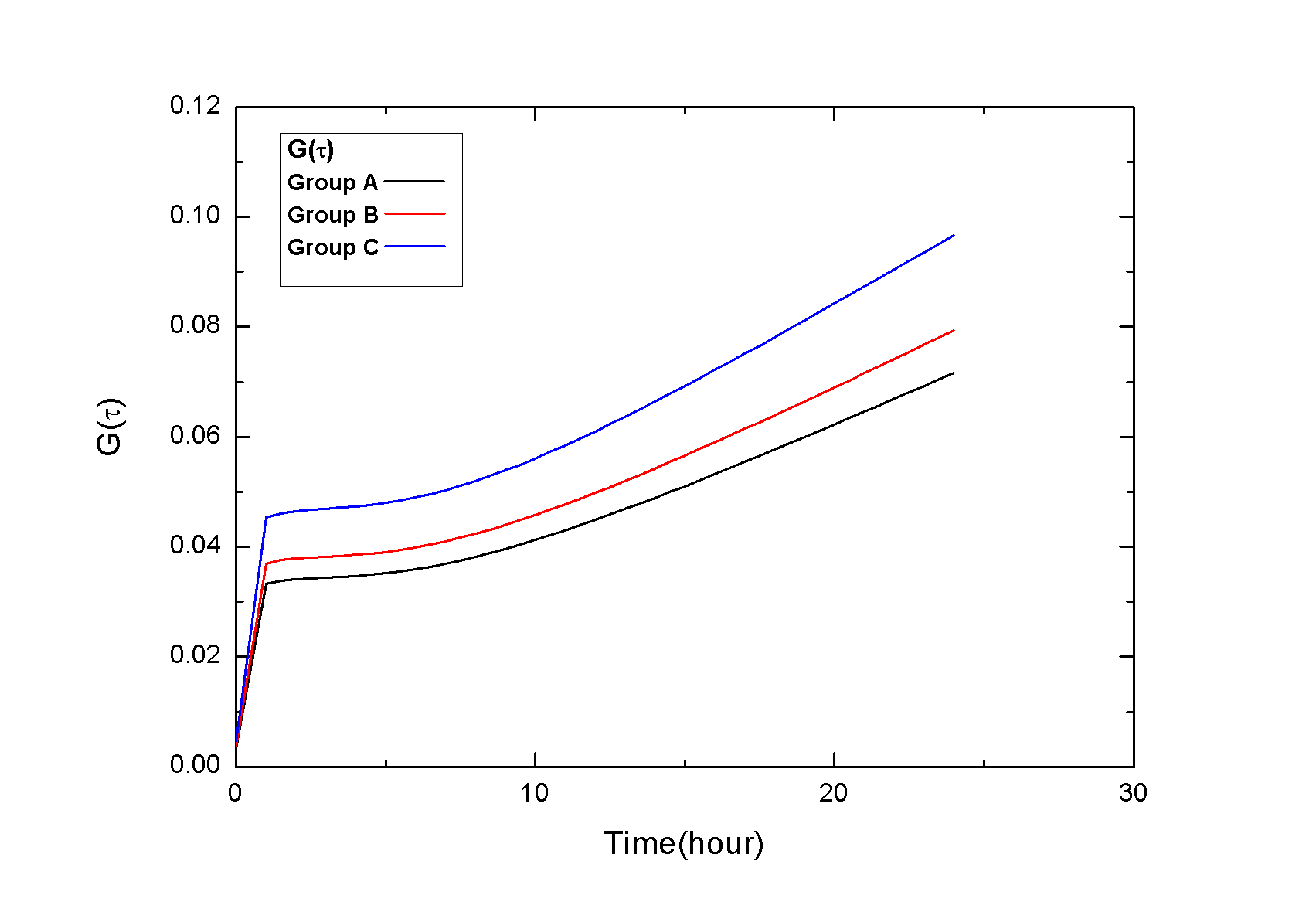

Supplement: Supplementary file 1 — Additional file 1. [file 13014_2020_1555_MOESM1_ESM.docx]
